# Anthropogenic pollution gradient along a mountain river affects bacterial community composition and genera with potential pathogenic species

**DOI:** 10.1038/s41598-022-22642-x

**Published:** 2022-10-28

**Authors:** Anna Lenart-Boroń, Piotr Boroń, Klaudia Kulik, Justyna Prajsnar, Mirosław Żelazny, Maria J. Chmiel

**Affiliations:** 1 Department of Microbiology and Biomonitoring, Faculty of Agriculture and Economics, University of Agriculture in Kraków, Adam Mickiewicz Ave. 24/28, 30-059 Kraków, Poland; 2 Department of Forest Ecosystems Protection, Faculty of Forestry, University of Agriculture in Kraków, 29 Listopada Ave. 46, 31-425 Kraków, Poland; 3 Jerzy Haber Institute of Catalysis and Surface Chemistry, Polish Academy of Sciences, Niezapominajek Str. 8, 30-239 Kraków, Poland; 4 Department of Hydrology, Institute of Geography and Spatial Management, Jagiellonian University in Kraków, Gronostajowa Str. 7, 30-387 Kraków, Poland

**Keywords:** Microbiology, Environmental sciences

## Abstract

Mountain regions in Poland are among the most frequently visited tourist destinations, causing a significant anthropogenic pressure put on the local rivers. In this study, based on numbers of 9 microorganisms, content of 17 antibiotics and 17 physicochemical parameters, we determined a pollution gradient in six sites along Białka, a typical mountain river in southern Poland. The *E.coli*/*Staphylococcus* ratio varied evidently between polluted and non-polluted sites, indicating that the possible utility of this parameter in assessing the anthropogenic impact on river ecosystems is worth further investigation. Then, using next generation sequencing, we assessed the changes in bacterial community structure and diversity as a response to the pollution gradient. *Proteobacteria* and *Bacteroidetes* were the most abundant phyla in the majority of samples. *Actinobacteria* were the most abundant in the most pristine (groundwater) sample, while *Firmicutes* and *Verrucomicrobia* were more prevalent in polluted sites. Bacterial diversity at various levels increased with water pollution. Eleven bacterial genera potentially containing pathogenic species were detected in the examined samples, among which *Acinetobacter*, *Rhodococcus,* and *Mycobacterium* were the most frequent. At the species level, *Acinetobacter johnsonii* was most prevalent potential pathogen, detected in all surface water samples, including the pristine ones. Two bacterial taxa—genus *Flectobacillus* and order *Clostridiales* showed very distinct variation in the relative abundance between the polluted and non-polluted sites, indicating their possible potential as biomarkers of anthropogenic impact on mountain river waters.

## Introduction

In 2021, Poland has been classified as the 24th (among 27) European Union (EU) country in terms of renewable sweet water resources, amounting to 1600 m^3^ per capita, while according to the United Nations Organization (UNO), 1700 m^3^ per capita is the limit, below which a country is considered as threatened by water scarcity^1^. Therefore, freshwater contamination, most importantly the deterioration of river water quality, is one of the greatest issues of water management in Poland. The fact that the vast majority of mountain areas in Poland are of rural character, where sewage treatment and disposal are at unsatisfactory level, makes mountain rivers particularly vulnerable to the detrimental effect of anthropogenic pressure. An example of such a river is Białka (Tatra mountain region, Southern Poland) that, despite its relatively short course, constitutes a significant resource for local community providing a number of services. These include recreation, irrigation, a drinking water source, general-use household water source, and finally the source of water for artificial snow production for the ski slopes along the river valley^2^. The Białka river is also a representative example of mountain rivers subjected to a variety of anthropogenic pressure-associated effects, among which increased water consumption and excess production of sewage associated with developed tourism intensity, have been proved to be the main issues^3^. The situation observed in the Białka river valley corresponds to the one observed in other mountain regions of the world^4–7^.

According to Godoy et al.^8^, the efficacy and intensity of microbial responses to the environmental changes largely depending on the functional properties and/or population size of bacterial community. Bacteria are the dominant and most diverse organisms in freshwater ecosystems and they play a key role in biogeochemical processes, including the removal of contaminants^9^. Microbial communities are very dynamic and they reflect the changes in ecosystem condition, e.g. by shifts in some of the bacterial taxa coupled with decrease in others as a result of inflow of a variety of contaminants^10,11^. Given the substantial effect that anthropogenic pressure has on water environments, it is of vast importance to obtain reliable and comprehensive information about the response of microbial communities within different types of water^12^. Mountain rivers are unique environments and very dynamic ecosystems with distinct and varying morphological, climatic, hydrological, hydrochemical and biological features. This makes them particularly susceptible to human interference as the sizes of these morpho-climatic zones are relatively small compared to lowland rivers. Even though a number of remote mountain rivers still seem intact, the majority of mountain ecosystems are being increasingly affected by the market economy-associated pressure^13^. With the common use of bacterial populations as the sensitive indicators of water quality and biomonitoring, gathering information about the structure and diversity of bacterial communities in mountain rivers expands our knowledge about this type of environment and increases our understanding of microbial ecology in mountain habitats. Thus, the aim of this study was to: (I) determine the pollution gradient along Białka, the typical mountain river, (II) examine the bacterial diversity and abundance in the water of the Białka river along the pollution gradient, and (III) assess the presence of bacterial genera containing potentially pathogenic species in the examined water samples along the pollution gradient of the Białka river.

## Results and discussion

This study analyzed the effect of varying anthropogenic pressure along the typical mountain river on the concentration of conventional bacterial indicators of water quality, the major ions, the antibiotic concentration as well as the bacterial community structure and diversity.

### Pollution gradient along the course of the Białka river

**Figure 1:**
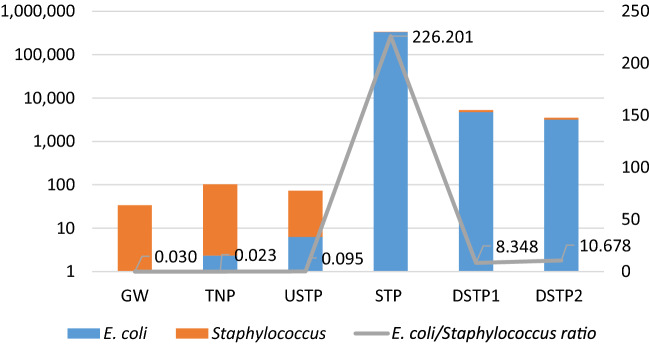
Mean concentrations (CFU/100 ml) of *E. coli* and *Staphylococcus* spp. in water, and changes in the *E.coli*/*Staphylococcus* spp. ratio along the course of the Białka river. Site abbreviations are as follows: GW—groundwater; TNP—Tatra National Park; USTP—upstream of the sewage treatment plant; STP—sewage treatment plant; DSTP1—c.a. 3 km downstream of the STP; DSTP2—c.a. 7 km downstream of the STP (for detailed description, please see caption of Fig. 2).

The study showed that the detected number of bacterial contaminants changes along the course of the Białka river and varies significantly, depending on the study site and anthropogenic pressure put thereon. The numbers of all culturable microorganisms observed in this study are provided in Supplementary Table S1. Figure 1 shows how the mean concentrations of *E. coli* and *Staphylococcus* spp. (CFU/100 ml) change from low in the groundwater and upper course of the river, then rise when reaching the first built-up areas (USTP), followed by a drastic increase by the sewage treatment plant, to drop slightly downstream of the STP (DSTP1 and DSTP2). What became evident (Fig. 1), is that the ratio between the typical indicator of fecal water contamination (*E. coli*) and *Staphylococcus* spp., whose presence in water might be of various origin (including surface runoff, human activity, point, and non-point sources of water contamination^14^), is very low in the upper course of the Białka river, then it dramatically increases by the STP (226.20) and drops to the values around 10 downstream of the STP. Having in mind that such a relationship has not been examined before, we suggest that the utility of the *E. coli*/*Staphylococcus* spp. ratio as an indicator of water contamination and the origin of contaminants is worth being subjected to further studies.

**Figure 2:**
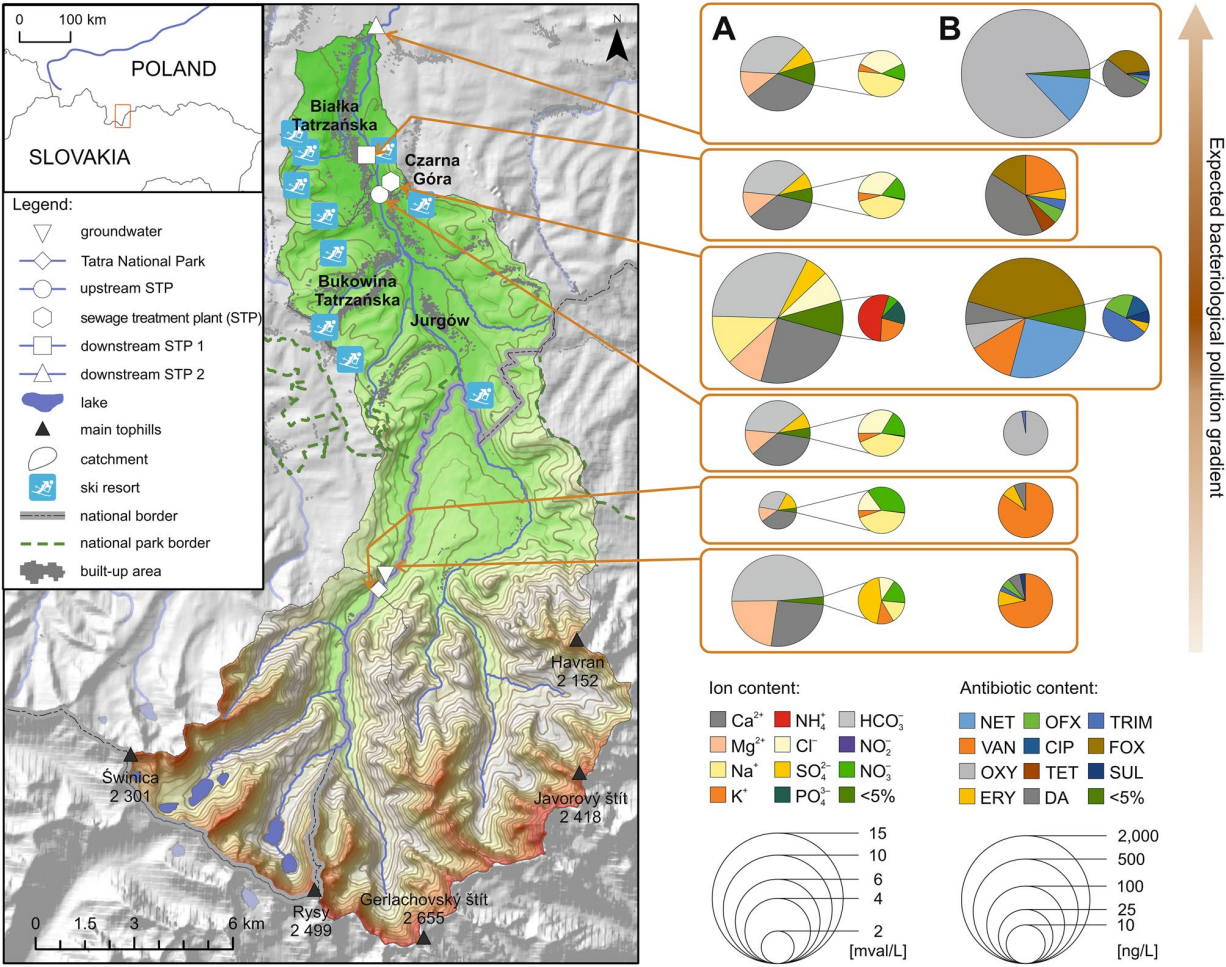
Study area and sampling sites along the Białka river. The 1st site is located at the border of the Tatra National Park (TNP) and is characterized by the cleanest waters. The 2nd site is situated c.a. 1 km upstream of the sewage treatment plant (USTP) of the Bukowina Tatrzańska municipality. The 3rd site is located by the effluent from the Bukowina Tatrzańska sewage treatment plant (STP). The 4th site is situated approximately 3 km downstream of the STP discharge, in the center of Białka Tatrzańska ski resort (DSTP1), and the 5th site—approximately 7 km downstream of the STP discharge in the Trybsz village (DSTP2). Groundwater collected in the Tatra National Park was treated as control, i.e. the most pristine water (GW)^3^. Pie chart graphs to the right show color coded ion (**A**) and antibiotic (**B**) content detected in particular sites during all three sampling dates. The size of the primary charts is logarithmically scaled (see the scales to the bottom right), the secondary charts have not been content-scaled. Please note significantly increased ion and antibiotic content in the Białka river after passing the STP site. Arrow graphs show the expected bacterial pollution gradient, darker brown shade indicates the more contaminated sites, the arrowhead indicates the course of Białka river.

Figure 2 presents the mean values of nutrient concentrations detected for all study sites along the Białka river. The total content of nutrients varies evidently between the surface water samples located along the Białka river. The surface water sample located in the Tatra National Park (TNP) is characterized by the lowest content of the examined ions (the total value of 80.80 mg/l), followed by a rise by the first households (USTP site – with the total nutrient content of 148.76 mg/l). Then, a dramatic increase can be observed at the sewage treatment plant (STP, total ion content of 578.18 mg/l), after which the nutrient content decreases downstream (DSTP1 with 163.03 mg/l), then again slightly increases after passing the Białka Tatrzańska and Trybsz villages (DSTP2 with 182.19 mg/l). The total content of ions in the groundwater samples (GW—217.01 mg/l) is higher than the concentrations observed upstream of the STP, but here Ca^2+^ and HCO_3_^-^ (which are typical predominant ions in groundwater of mountainous regions^15^) have dominant share in the total ion content.

Similarly as in the case of culturable bacteria and nutrient concentration, the content of antibiotics in water (Fig. 2, Supplementary Table S2) is either none, or very low in the Tatra National Park or upstream of the STP. A remarkable increase by the sewage treatment plant can be observed, followed by a significant decrease c.a. 3 km downstream of the STP (DSTP1), after which the antibiotic content in water increases again after the water flows through the villages (DSTP2). The fact that the neighboring municipal sewage treatment plant and its poor infrastructure serves as the local hotspot for antibiotic substances dissemination in the water environment, has been described and discussed in one of our previous papers^16^. Moreover, it is a known issue that sewage treatment plants are among the main receptors of antibiotics which then are released into the environment, including river waters, and it has been reported by a number of researchers^17,18^. In this study, as many as 11 antibiotic substances out of 17 examined were detected (Supplementary Table S2). Erythromycin (macrolides), clindamycin (lincosamides), and trimethoprim (dihydrofolate reductase inhibitors) were detected most frequently (in more than 50% of samples), whereas the highest concentrations were observed for the following three antibiotics: oxytetracycline (tetracyclines; 1750 ng/l in DSTP2), netilmicin (aminoglycosides; 315 ng/l in STP and 242 ng/l in DSTP2), and cefoxitin (cephalosporins; 261 ng/l and 255 ng/l in STP) (Fig. 4). When comparing the results obtained in our study, with the ones focused on comprehensive and multi-class determination of pharmaceuticals in water and wastewater samples^17,19,20^, it can be seen that trimethoprim, sulfamethoxazole, ciprofloxacin, and clindamycin are the antimicrobial agents detected in all studies. Similarly as in the study by Rodriguez-Mozaz et al.^17^, the 1:5 ratio between the concentrations of trimethoprim and sulfamethoxazole, the combination of which is commonly used in clinical treatment^21^, was not reflected in the concentrations of these two compounds in water. Trimethoprim, sulfamethoxazole, and ciprofloxacin, which were detected in this study in 56%, 17% and 6% of studied samples, respectively, have been included in the most recent watch list of substances for the European Union-wide monitoring, due to the fact that these substances have been assessed to pose a significant risk to aquatic environment^22^.

With respect to the examined groups of parameters, i.e. bacterial indicators of water quality, nutrient, and antibiotic concentrations in the sites located along the Białka river, it can be clearly seen that the varying level of water pollution can be linked to the increasing anthropogenic pressure along the river valley. The overall gradient of water pollution can be summarized as: GW < TNP < USTP < DSTP1 < DSTP2 < STP. One may assume that the water contamination should decrease with increasing distance from the STP, due to the naturally occurring self-purification process^23^. However, due to the fact that the Białka river flows through highly impacted region, a number of point sources of river contamination can be found along the course of the river, particularly in its lower reaches^24^.

In the following parts of the paper we tried to assess whether this changing pollution gradient affects the changes in bacterial community structure, its diversity as well as the compositional and functional changes, as obtained from the NGS data.

### Changes in the bacterial community structure and diversity along the course of the river / pollution gradient of the Białka river

**Figure 3:**
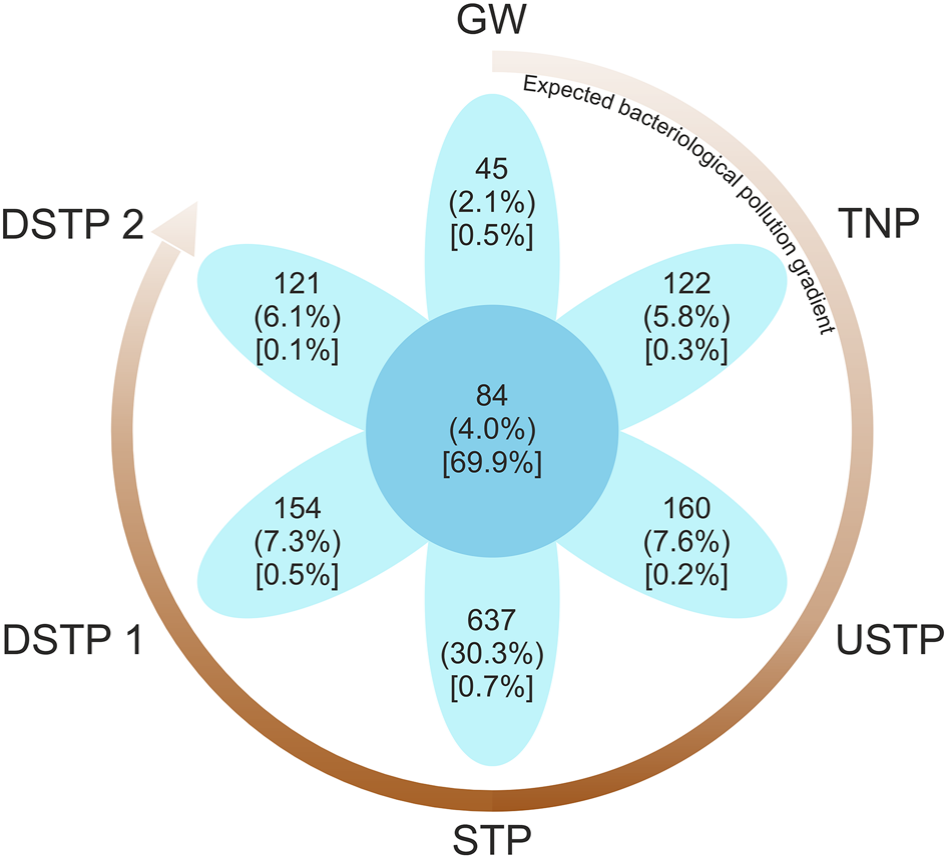
Venn diagram showing bacterial genera unique to and shared between the examined sites along the Białka river. Values in petals indicate from the top: numbers of genera in each group; proportions of the overall number of genera in each group (in round brackets), and proportions of 16S sequences belonging to unique genera in each group (in square brackets). Arrow graph shows the expected bacterial pollution gradient, darker brown shade indicates the more contaminated sites, the arrowhead indicates the course of Białka river. Please note the diversity increase similar to the pollution gradient, i.e. GW < TNP = DSTP2 < USTP = DSTP1 < STP, expressed by high numbers / proportion of unique genera, more diverse composition of bacterial genera in more polluted sites. Site abbreviations are as follows: GW—groundwater; TNP – Tatra National Park; USTP—upstream of the sewage treatment plant; STP—sewage treatment plant; DSTP1—c.a. 3 km downstream of the STP; DSTP2—c.a. 7 km downstream of the STP (for detailed description, please see caption of Fig. 2).

There is a variety of bacterial community structure, richness, and diversity measures, including the number of operational taxonomic units (OTUs), the number of unique OTUs, as well as Shannon and Simpson indices^25^. As can be observed in Fig. 3 (petal diagram constructed for the bacterial genera unique to and shared between the examined sites), the overall number of genera and the share of genera unique to individual sites were the lowest in the groundwater (GW), i.e. the most pristine sample, and those numbers increased following the pattern of the pollution gradient suggested above. This observation is consistent with the tendency presented by e.g. de Oliveira and Margis^28^, where the river water samples from the less impacted regions were characterized by smaller alpha-diversity measures as compared to the samples from the areas most impacted by human activity.

**Figure 4:**
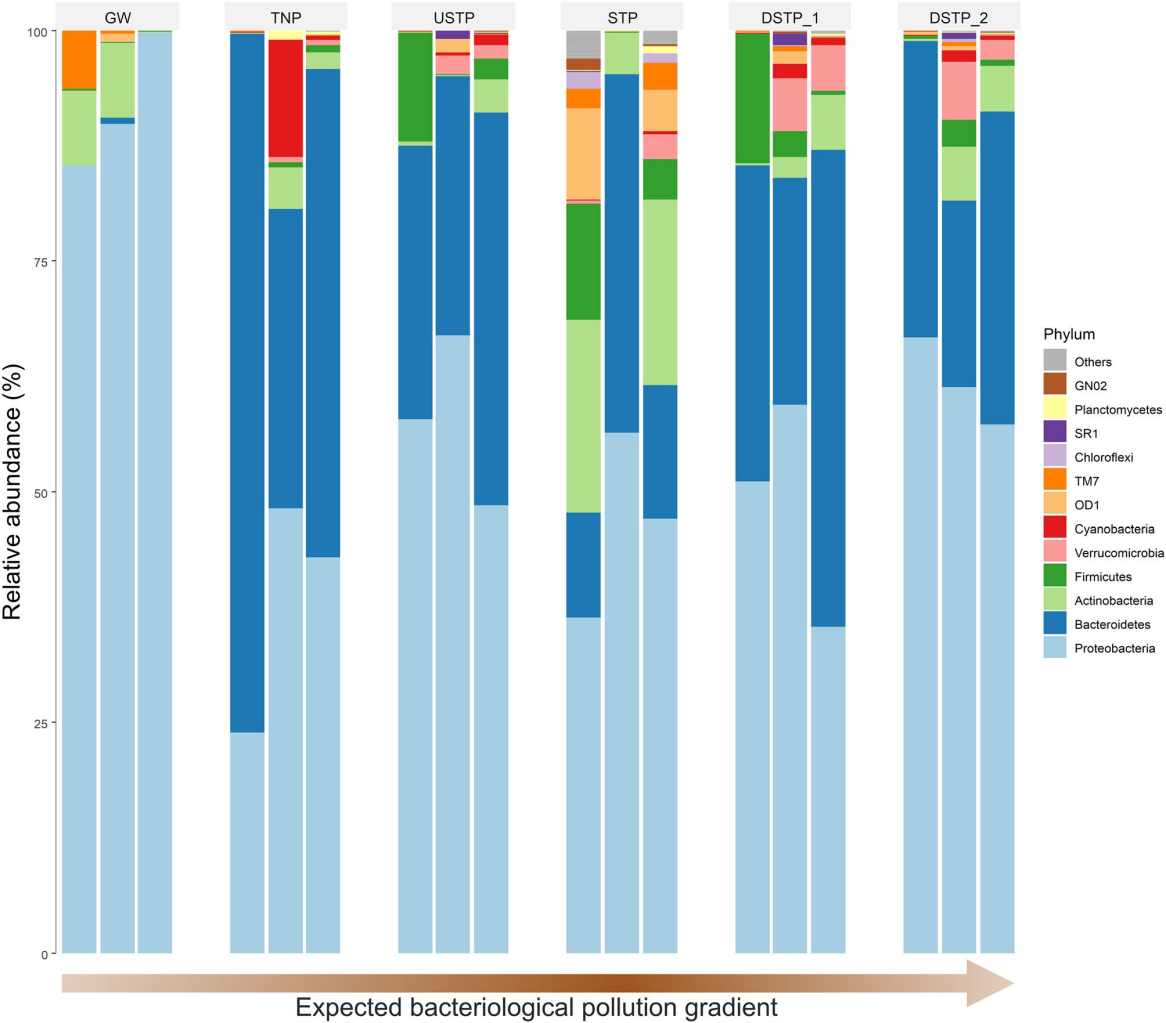
Changes in the relative abundance of bacteria at the phylum level along the pollution gradient of the Białka river. Three sampling seasons, i.e. summer, winter, and spring are shown from left to right. Arrow graph shows the expected bacterial pollution gradient, darker brown shade indicates the more contaminated sites, the arrowhead indicates the course of Białka river. Site abbreviations are as follows: GW—groundwater; TNP—Tatra National Park; USTP—upstream of the sewage treatment plant; STP—sewage treatment plant; DSTP1—c.a. 3 km downstream of the STP; DSTP2—c.a. 7 km downstream of the STP (for detailed description, please see caption of Fig. 2).

As many as 99.94% of identified OTU reads from the examined samples were assigned to 22 phyla, among which the 12 most prevalent in all samples are shown in Fig. 4. *Proteobacteria* was the most abundant phylum in all samples. The second most abundant phylum in all surface water samples was *Bacteroidetes*, while in the case of groundwater, *Actinobacteria* were the second most abundant phylum. *Actinobacteria* are commonly found in groundwater^26^ and were observed in relatively higher abundances in groundwater samples than in aquifer water samples by Korbel et al.^27^. Generally, the dominance of *Proteobacteria*, *Bacteroidetes*, and *Actinobacteria* has been observed in freshwater environments, including river waters^8^, de Oliveira and Margis^28,29^. The phylum *Verrucomicrobia* was absent or its numbers were negligible in both clean sites (GW and TNP), throughout the study. *Bacteroidetes* was abundantly present only in all surface water samples, and absent/negligible in GW. Members of *Bacteroidetes* were reported by various authors as abundantly present in water affected by anthropogenic pollution^30,31^. However, the predominant genus within this phylum among the surface water samples was *Flavobacterium* (Supplementary Fig. S1), which is typically found in various freshwater environments, rather than in human gastrointestinal tract^32^. The relative abundance of both, *Verrucomicrobia* and *Firmicutes*, exceeded the level of 1% in the sites USTP, and reached their maximum levels either by the STP, or in the sites located further along the river (DSTP1 and DSTP2). The two sites most abundant in *Firmicutes* were DSTP1 and STP, where their relative abundance reached 14.05% and 12.49%, respectively. On the other hand, the relative abundance of *Verrucomicrobia* was the highest in DSTP2 and DSTP1 (6.28% and 5.69%, respectively). Increasing abundance of *Verrucomicrobia* associated with watershed urbanization (and thus – pollution of water) was also observed by Lin et al.^33^. Numerous *Firmicutes* members are the typical human gut microbiota^34^ whose main source in river water is sewage inflow. Similarly to our results, the increased numbers of *Firmicutes* resulting from anthropogenic sources were reported in the Yangtze river by Sun et al.^35^. In general, high relative abundance, or even a dominant position, of *Firmicutes* has been reported as an important indication of fecal pollution^29,36^.

What can be observed from Fig. 4 and Supplementary Fig. S1, is that the composition of bacterial communities in surface water samples is very variable (with differences clearly seen both between the sampling sites and between seasons of the year). In contrast, the groundwater (GW) sample is characterized by the smallest variation at the level of phylum (Fig. 4) or genus (Supplementary Fig. S1). This observation is also supported by the lowest species evenness and the lowest Shannon diversity index calculated for this site^3^. The described situation is similar to that observed by de Oliveira and Margis (2015), who stated that microbial populations are maintained by a core of OTUs that persist longitudinally and seasonally. They observed that despite the differences among seasons and the course of the river, the main core of bacterial community is maintained. It was suggested that the river’s source continuously restores the microbial diversity along the river course. These findings emphasize the importance of the preservation of river resources, including the purity of the rivers’ sources as one of the ecological keys for the recovery and maintenance of rivers affected by anthropogenic factors (Oliveira and Margis 2015).

### Compositional differences in bacterial communities as a response to the pollution gradient of the Białka river

**Figure 5:**
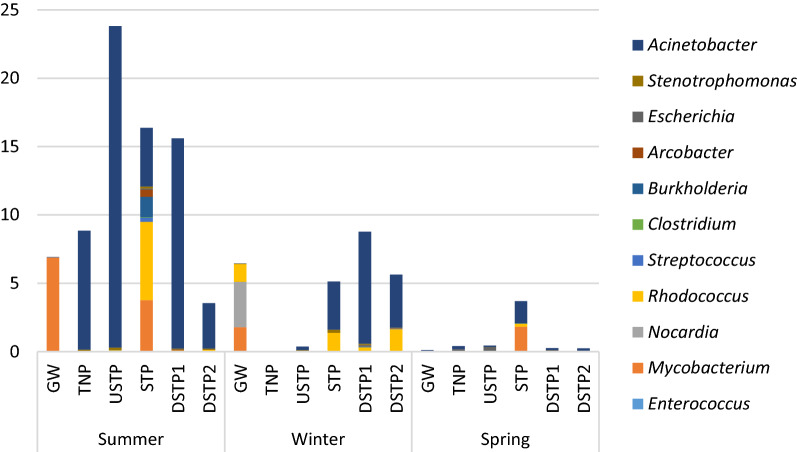
Bacterial genera with potentially pathogenic species, the relative abundance of which in the studied sites exceeded 0.01% Site abbreviations are as follows: GW—groundwater; TNP—Tatra National Park; USTP—upstream of the sewage treatment plant; STP—sewage treatment plant; DSTP1—c.a. 3 km downstream of the STP; DSTP2—c.a. 7 km downstream of the STP (for detailed description, please see caption of Fig. 2).

Based on the criterion of at least one species being categorized at biosafety level 2 or 3 within a genus, whose relative abundance exceeded 0.1% (see the Materials and Methods section), 11 bacterial genera containing potential pathogens were found in the examined samples (Supplementary Table S2, Fig. 5). Among them, the three most prevalent ones were *Acinetobacter* (detected in 15 out of 18 samples with the highest relative abundance of 23.53% observed in USTP site in summer), *Rhodococcus* (detected in 14 samples with the highest relative abundance of 5.73% in STP site in summer) and *Mycobacterium* (detected in 13 sites with the highest relative abundance of 6.90% in GW site also in summer). The very high abundance ratios of *Acinetobacter* was also reported by Ghaju Shrestha et al.^37^, who observed these bacteria in all samples with the relative abundances reaching even 63% in well waters, 34% in spring waters, and 31% in river water samples. What is more, the detection of *Acinetobacter* in high percentage of samples and its high relative abundance is worth paying attention to, because bacteria within this genus are reported throughout the world, not only as opportunistic and nosocomial pathogens with multi-drug resistance, but the presence of antimicrobial-resistant *Acinetobacter* strains (e.g. *A. johnsonii*, *A. baumanii*, *A. junii* and *A. calcoaceticus*) has also been reported in groundwater^38^. Having in mind the fact that the representatives of the genus *Acinetobacter* have been frequently reported in surface and groundwater samples^37–40^, the surveillance of these bacteria, with attention paid to the detection of obligatory pathogenic species, should be considered a part of the detection of microbial contamination of water resources. What is also interesting about the results shown in Fig. 5 and Supplementary Table S2, is the occurrence of potentially pathogenic *Mycobacterium*, *Nocardia*, and *Rhodococcus* in the groundwater (GW) sample in two seasons (summer and winter). The presence of these three species in groundwater was also reported by^41^, but the examined sample was collected from a borehole in a hydrocarbon-contaminated site, where a wide range of chemical compounds had been manufactured. Species identification of the OTU reads allowed for the detection of 13 species of bacteria categorized at 2 biosafety level according to American Biological Safety Association^42^. Among these, only two species, i.e. *E. coli* and *Acinetobacter johnsonii* were detected in the number of 100 reads or more (Table 1). These samples included: USTP (145 reads of *E. coli* and 1258 reads of *A. johnsonii*), STP (453 reads of *A. johnsonii*, DSTP1 (1680 reads of *A. johnsonii*) and DSTP2 (330 reads of *A. johnsonii*). At least two of the mentioned pathogenic species were detected in each of the examined samples, including GW (two species: *E. coli* and *Propionibacterium acnes*) and TNP (nine species: *Acinetobacter lwofii*, *A. johnsonii*, *E. coli*, *Stenotrophomonas maltophilia*, *Plesiomonas shigelloides*, *Bacillus cereus*, *P. acnes*, *Serratia marcescens* and *Myroides odoratum*). The GW and TNP samples in our study are derived from a pristine environment, i.e. Tatra National Park, which suggests that even presumably uncontaminated water sources may harbor potentially pathogenic microorganisms. However, in order to confirm that there is a cause for concern from the public health perspective, more detailed analyses are necessary. In this sense our results are preliminary and should be treated as an early warning. High-confidence detection of individual pathogens require more detailed methods than 16S V3-V4 barcoding, such as qPCR targeting individual species within the mentioned genera. However, despite its much higher resolving power, the detection of particular pathogens using qPCR method is not guaranteed due to its certain limitations, especially the need for high quality material, which may prove unsuccessful in environmental samples^8,43^. Moreover, high cost and labor-intensity of qPCR screening mean that usually only limited number of potential pathogens can be practically surveyed. Supplementary Table S2 summarizes the presence of bacterial genera potentially containing pathogenic species along with the gradient of water pollution in terms of bacterial indicators of water quality and the presence of antimicrobial agents. Based on the data presented in Table 1 and Supplementary Table S2 it can be stated that the sample collected at the STP is characterized by a variety of both bacterial genera containing potentially pathogenic species and the pathogenic species themselves. Such a result is not surprising, as the highly increased abundance and diversity of bacterial pathogens in feces-contaminated water has been often reported. For example, Ghaju Shrestha et al.^37^ observed the highest abundance and diversity of potential pathogens in water samples contaminated with human and animal feces, which most probably is also the case in our study.

**Table 1:** Pathogenic species of bacteria and their number of reads detected in the water samples along the pollution gradient of the river in different sampling dates.

Species	Sample						
	Pathogenicity	GW	TNP	USTP	STP	DSTP1	DSTP2
*Acinetobacter johnsonii*	Nosocomial infections (bacteremia, pneumonia, meningitis)	0	64	1258	453	**1680**	330
*Acinetobacter lwoffii*	Nosocomial infections (bacteremia, pneumonia, meningitis)	0	2	**41**	0	0	0
*Arcobacter cryaerophilus*	Enteropathogen (gastroenteritis) and potential zoonotic agent transmitted by food and water	0	0	0	**63**	6	2
*Escherichia coli*	Variety of diarrheas, urinary tract infection, abdominal and pelvic infection, pneumonia, bacteremia, and meningitis	27	68	**145**	23	41	18
*Stenotrophomonas maltophilia*	Meningitis, urinary tract infection, and wound infections intrinsically resistant to beta lactams, aminoglycosides, and quinolones as well as carbapenems	0	2	8	9	**12**	2
*Plesiomonas shigelloides*	Enteropathogen (gastroenteritis)	0	20	**58**	0	79	2
*Bacillus cereus*	Foodborne pathogen causing gastroenteritis, eye infections, pneumonia	0	**68**	0	2	0	0
*Propionibacterium acnes*	Acne as well as postoperative and device-related infections (infections of the bones and joints, mouth, eye and brain)	15	10	**34**	0	0	0
*Serratia marcescens*	Urinary, respiratory, and biliary tract infections, peritonitis, wound infections, and intravenous catheter-related infections, bacteremia	0	20	**22**	0	0	0
*Myroides odoratimimus*	Bacteremia and soft tissue infections in immunocompromised patients	0	6	2	2	**16**	10
*Bacteroides uniformis*	Intra-abdominal infections	0	0	0	**29**	0	0
*Lactococcus garvieae*	Enteropathogen (gastroenteritis); bacteremia in immunocompromised patients	0	0	0	**8**	2	0
*Veillonella parvula*	Osteomyelitis, bacteremia	0	0	0	**13**	0	0
	Total number of identified pathogens	2	9	8	9	7	6

Site abbreviations are as follows: *GW* groundwater, *TNP* Tatra National Park, *USTP* upstream of the sewage treatment plant, *STP* sewage treatment plant, *DSTP1* c.a. 3 km downstream of the STP, *DSTP2* c.a. 7 km downstream of the STP (for detailed description, please see caption of Fig. 2).

Information on pathogenicity was obtained from the following sources: Murray et al.^44^, Szewczyk^45^, Smith et al.^46^, Endicott-Yazdani et al.^47^; Fisher and Denison^48^.

The highest values are in bold.

**Figure 6:**
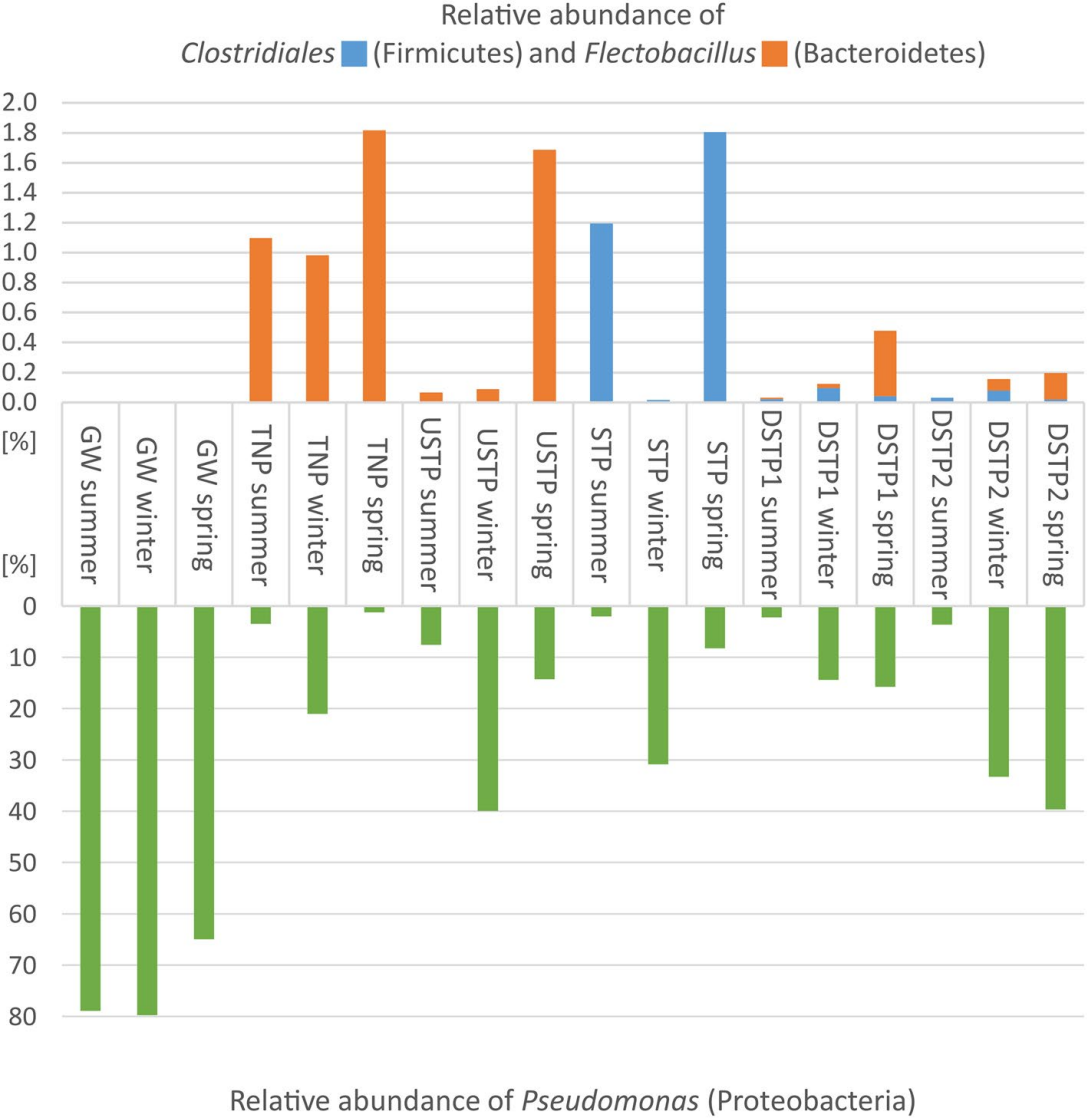
Variation in the most differentially abundant OTUs (% relative abundance) across the examined sites along the Białka river. Site abbreviations are as follows: GW—groundwater; TNP—Tatra National Park; USTP—upstream of the sewage treatment plant; STP—sewage treatment plant; DSTP1—c.a. 3 km downstream of the STP; DSTP2—c.a. 7 km downstream of the STP (for detailed description, please see caption of Fig. 2).

The discriminant analysis was applied to find out whether there are microbial taxa, the relative abundance of which differs between the examined groups of sites. Three taxa were selected (Fig. 6) as the ones that could be determined as taxonomic biomarkers showing the effect of anthropogenic pressure put on the mountain river, Białka. The members of phylum *Bacteroidetes* (genus *Flectobacillus*), showed clear differences in the relative abundance between the anthropogenically non-disturbed surface waters (high prevalence in TNP and USTP) and the sites impacted by anthropogenic activity. The relative abundance of these bacteria follows the pattern of the suggested pollution gradient along the Białka river with increasing abundance in more intact samples, i.e. STP > DSTP2 > DSTP1 > USTP > TNP. The members of this Gram-negative genus are not pathogenic and are highly susceptible to numerous antibiotics^49^. In the case of aquatic environments, *Flectobacillus* spp. have been found in freshwater springs^49^ and in rivers^50^. Another genus, the relative abundance of which differentiated the examined water samples, was *Pseudomonas*, phylum *Proteobacteria*. Its highest relative abundance was observed in groundwater (GW), whereas among the surface water samples, its prevalence did not differ significantly. The order *Clostridiales* (phylum *Firmicutes*) was found to be distinctly more prevalent by the sewage treatment plant (STP site) rather than in other sites. The members of *Clostridiales* are among major indicators of fecal contamination and have been found in odorous rivers with extremely low oxygen content^51^. The high prevalence of *Clostridiales* demonstrates degraded environmental quality of a river environment^52^. Shang et al.^52^, in their study conducted on a 97-km Nanjing reach of the Yangtze river, suggested that the environmental stress put on the aquatic microbial community ultimately shaped its bacterial diversity. After thoroughly examining the interspecies interactions, they suggested that the varying prevalence and interactions between members of certain bacterial orders (reportedly: *Clostridiales*, *Nitrospirales* and *Myxococcales*) may serve as an indication of the river’s ecological health. In our study, high relative abundance of *Clostridiales* was associated with anthropogenically impacted sites (STP, DSTP1, DSTP2), while the sites non-impacted by anthropogenic activity (GW, TNP, USTP) were characterized by the lack of these bacteria (Fig. 6). On the other hand, *Flectobacillus* spp. were more abundantly observed in the non-polluted sites compared to the polluted ones (Fig. 6). Spearman’s rank correlation coefficients (Supplementary Table S3) indicate that the occurrence of genus *Flectobacillus* correlated negatively with the concentrations of cefoxitin (average correlation, −0.479, *p* > 0.005) and with Ca^2+^, Mg^2+^, and K^+^ ions. Also the genus *Pseudomonas* and members of the family *Pseudomonadaceae* correlated negatively with cefoxitin (−0.543, *p* > 0.005), suggesting that this antibiotic agent effectively reduces the populations of these bacterial taxa. Conversely, various members of the order *Clostridiales* (e.g. *Megamonas*, *Ruminococcus*, *Dorea*, *Faecalibacterium*, *Anaerosinus*) correlated positively (r > 0.5) with a number of antimicrobial agents, such as oxytetracycline, trimethoprim, ofloxacin, clindamycin, cefoxitin, and sulfamethoxazole. Most of these taxa are associated with human gut microbiota and/or have been found in feces, showing that the detected antimicrobial agents originated from sewage. Changes in the bacterial community composition, as a result of the selective pressure posed by antibiotics on some bacterial taxa, is a known phenomenon. Guan et al.^53^, for instance, observed that trimethoprim, sulfamerazine and oxytetracycline selectively promoted the growth of some opportunistic pathogens, such as *Aeromonas*, *Acinetobacter* and *Arcobacter*, while Li et al.^54^ reported a similar situation to our study, that is the positive correlation between *Clostridia* (e.g. *Clostridium*, *Ruminococcus*) and the level of river water contamination with antibiotics such as penicillin and oxytetracycline. Tong et al.^55^, on the other hand, in their study on the effect of ofloxacin and tetracycline on bacterial communities in constructed wetlands observed that the relative abundance of *Pseudomonas* decreased in response to the presence of the two examined antibiotics. Moreover, a strong positive correlation was observed between the presence of *Clostridiales* and NH_4_^+^, NO_2_^-^, NO_3_^-^, Na^+^, and K^+^ ions, which are associated with low water quality resulting from anthropogenic impact^24,56^. Observations of this type may help better understand the condition of riverine environments and may suggest the directions for the actions aimed at river health recovery. However, further exploration of the microbial community responses to environmental stress is certainly required.

**Figure 7:**
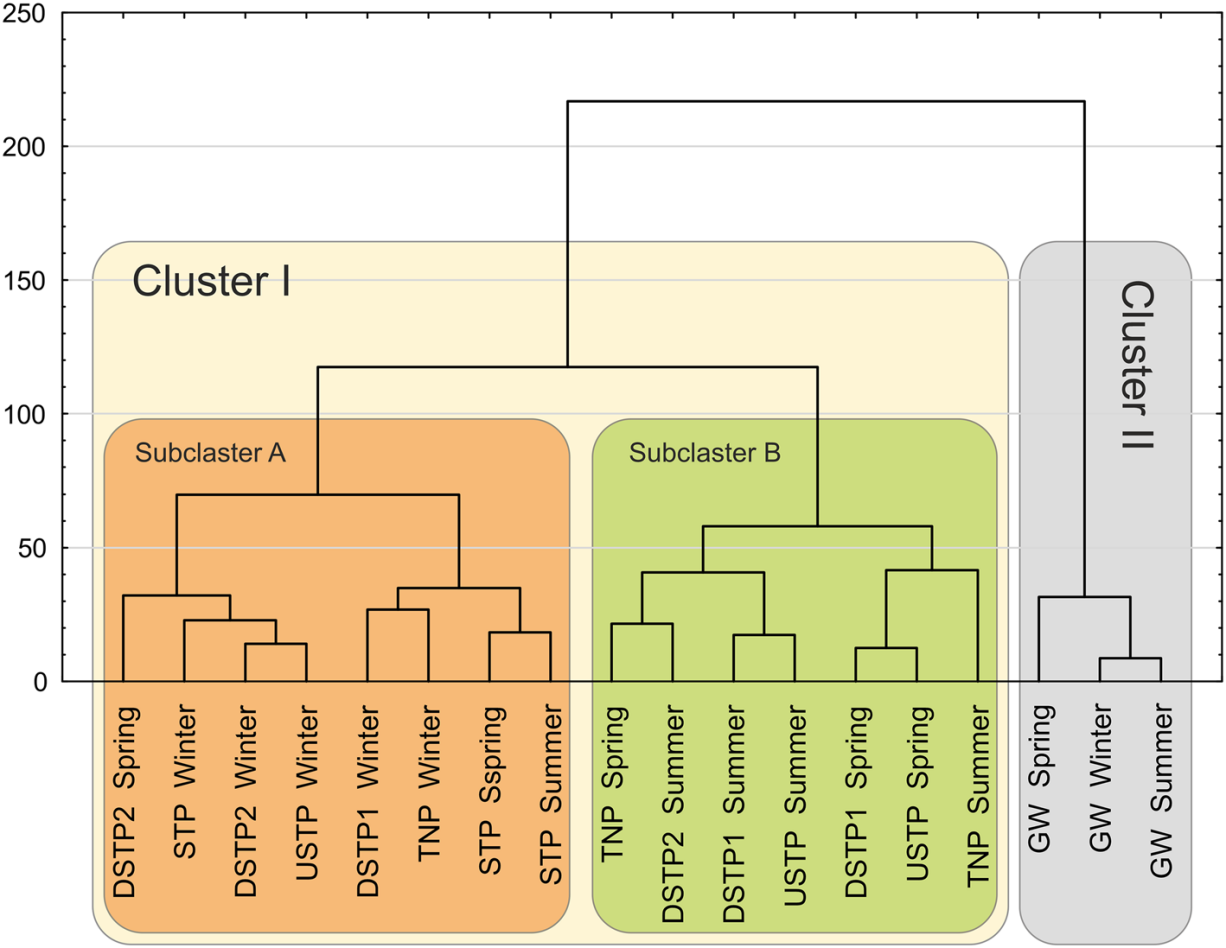
Cluster analysis of the studied sites based on the relative abundance of the bacterial genera. Site abbreviations are as follows: GW—groundwater; TNP—Tatra National Park; USTP—upstream of the sewage treatment plant; STP—sewage treatment plant; DSTP1—c.a. 3 km downstream of the STP; DSTP2—c.a. 7 km downstream of the STP (for detailed description, please see caption of Fig. 2).

The Cluster Analysis (Fig. 7) showed a clear distinction of the groundwater samples (GW) from the samples of surface water along the river. The clustering pattern of the river samples was correlated with the seasonal differences rather than with the spatial pollution gradient along the Białka river. Cluster I is divided into two sub-clusters (A and B), with no evident distinction of spatial pattern. Sub-cluster A groups all winter samples along the river, while sub-cluster B groups the majority of summer samples (except from STP). As for the spatial grouping, the only regularity is that the sub-cluster A groups all STP samples. In terms of the seasonal pattern, our results correspond to the ones obtained by Ma et al.^57^ in their study conducted on anthropogenically disturbed Haihe river, who also observed an apparent seasonal variation among the riverine samples. However, apart from clear seasonal clustering of the Haihe river samples, Ma et al.^57^ also observed that the spring samples formed three—urban, rural, and estuary—clusters, attributing the observed differences to the anthropogenic impact and water pollution. What can be observed in Fig. 7, is that the sub-cluster A of cluster I groups all winter and all STP, i.e. the most heavily polluted, samples. This may result from the fact that the mountain regions in Poland undergo very noticeable seasonal changes in the intensity of tourist traffic and, therefore, the river pollution is also impacted by this seasonality^3,24^. As a result of increasing popularity of skiing, Polish mountain regions, including the Białka river valley, became crowded by vast numbers of tourists, what exceeds the capacity of the local sewerage system, thus contributing to the contamination of the mountain river water^24^. However, as demonstrated in our previous study, this tourist traffic-related disturbance of the aquatic environment is not irreversible^3^.

## Concluding remarks

This study presents a thorough examination of changes in bacterial community structure of a typical mountain river, such as Białka, in response to the varying levels of pollution due to anthropogenic activity. A number of parameters, such as the presence of microbial indicators of water quality (e.g. coliforms, *E. coli*, *E. faecalis* or *Staphylococcus* spp.), ion concentrations, and concentrations of antibiotics in the water samples demonstrated that the surface water pollution clearly varies along the course of the river and this variation is reflected in the bacterial community structure changes. Also, conspicuous differences in *E. coli*/*Staphylococcus* spp. ratio along the course of the Białka river were found, suggesting that this ratio (high in anthropogenically impacted sites and low in less impacted sites) might be worth further investigation as the potential marker of the anthropogenic disturbance of the river ecosystem.

The alpha-diversity of aquatic bacterial community is noticeably affected by the anthropogenic impact on the river environment. A clear dominance of bacterial phyla characteristic of freshwater was observed in the non- or less impacted water samples, whereas the fecal contamination of river water resulted in the dominance of bacterial phyla typical of the contaminated sites. Thus, the pollution gradient along the Białka river shaped the compositional properties of its bacterial community. Among the 263 identified genera, 11 were recognized as those containing potentially pathogenic species with their highest numbers observed at the sewage treatment plant. *Acinetobacter*, *Rhodococcus*, and *Mycobacterium* were the most abundant bacterial genera potentially containing pathogenic species. Out of the identified potentially pathogenic species (species-level identification using 16S V3-V4 barcode) *Acinetobacter johnsonii* was the most abundant one and was detected in all samples except groundwater. The STP discharge was identified as the main source of environmental and public health concern along the river, but even the samples considered as uncontaminated may be the source of potentially pathogenic microorganisms. The relative abundance and frequency of detection of these bacteria corresponded to the suggested pollution gradient of the river. Furthermore, two bacterial taxa, i.e. *Clostridiales* (phylum *Firmicutes*) and *Flectobacillus* (phylum *Bacteroidetes*) showed evident differences in relative abundance, distinguishing between the most polluted (STP) and the least (TNP) polluted sites. With respect to the listed observations, it may be reasonable to consider that simply measuring the numbers of CFUs of bacterial indicators of water quality may not be the accurate and sufficient diagnostic method to assess the actual bacteriological threat as well as to track changes and disturbance rate as a result of the anthropogenic activity.

## Methods

### Sampling site and sample collection

The sampling sites were selected based on 7 years of experiments conducted along the Białka river valley^3,24^. Six sites, most representative in terms of the varying anthropogenic pressure, were selected along the course of the river (Fig. 2). The water samples were collected in three dates: August 2019 (summer tourist season), early March (late winter tourist season), and in May 2020 (spring, during COVID-19 lockdown^3^).

The samples of water for Illumina sequencing, for determination of antibiotic content and for microbiological analyses were collected into 1 l sterile polypropylene bottles; for the determination of hydrochemical parameters—into 500 ml polyethylene bottles. Water temperature (T), electrolytic conductivity (EC_25°C_), and pH were measured onsite during sampling with a handheld multimeter (YSI Pro 2030; USA).

### Enumeration, isolation, and identification of culturable bacteria

The membrane filtration method was used to enumerate *Escherichia coli*, coliforms and *Enterococcus faecali*s in water, while mesophilic and psychrophilic bacteria, *Staphylococcus* spp., *Salmonella*, *Shigella*, and microscopic fungi were enumerated using serial dilutions method, as described in Lenart-Boroń et al.^3^, Lenart-Boroń et al.^16^, and Lenart-Boroń et al.^24^. *E. coli* was grown on Tryptone Bile-X-glucuronide agar (TBX) agar and incubated at 44 °C for 48 h. Blue-green colonies, preliminarily identified as *E. coli* were then purified by plate streaking and their species was confirmed by MALDI-TOF mass spectrometry. For coliforms, purple red colonies with metallic sheen were counted on Endo agar (after incubation at 37 °C for 48 h), for *E. faecalis*—dark red to light brown colonies on Slanetz-Bartley agar (after incubation at 37 °C for 72 h). Mesophilic and psychrophilic bacteria were enumerated on Trypticase Soy Agar (after incubation at 37 °C for 48 h for mesophilic bacteria and at 4 °C for 72 h for psychrophilic bacteria), *Staphylococcus* spp. was enumerated on Chapman agar (incubation at 37 °C for 48 h), *Salmonella* and *Shigella* on SS agar (incubation at 37 °C for 48 h) while microscopic fungi on malt extract agar (MEA, incubation at 22 °C for 5–7 days). After incubation, visible colonies were counted and the results were expressed as the number of colony forming units per 100 ml (CFU/100 ml).

### Hydrochemical analyses of water

Ion chromatography was used to determine the concentration of Ca^2+^, Mg^2+^, Na^+^, K^+^, HCO_3_^−^, SO_4_^2−^, Cl^−^, NH_4_^+^,NO_3_^−^, NO_2_^−^, PO_4_^3−^, Li^+^, Br^−^, F^−^). A DIONEX ICS-2000 chromatograph and an AS-4 autosampler (Dionex, Sunnyvale, USA) were used, as described previously^3^.

### Determining the concentration of antibiotics in water

The main rationale for the selection of antibiotics for the analysis was their wide application in human and veterinary medicine on a global and national scale (European Centre for Disease Prevention and Control 2019^58^; European Medicines Agency 2018^59^). The examined antibiotics included ampicillin, amoxicillin, cefoxitin, ceftazidime, cefuroxime, clindamycin, doxycycline, erythromycin, gentamicin, netilmicin, ofloxacin, oxytetracycline, piperacillin, sulfamethoxazole, tetracycline, trimethoprim, and vancomycin.

Solid phase extraction (SPE) cartridges (Oasis HLB 6 cc Vac Cartridge, 500 mg sorbent per cartridge, 60 μm particle size, Waters, Milford, USA) were used to extract antibiotics from water samples. Each cartridge was first conditioned using 10 ml of methanol and three times with 5 ml of ultrapure water at a flow rate of 1–2 ml/min. One l of pre-filtered water sample (0.45 μm Sartorius filters) was then passed through the SPE cartridge at a flow rate of 10–20 ml/min., after which the SPE cartridges were dried under vacuum pressure for 30 min and the compounds eluted with 10 ml of methanol at a flow rate of 1–2 ml/min. Finally, the samples were completely dried and dissolved in 1 ml of methanol^60^.

An Ultra High Performance Liquid Chromatography (UHPLC) device equipped with an automatic autosampler (Agilent 1290 Infinity System) and mass spectrometer (MS) Agilent 6460 Triple Quad Detector (Santa Clara, USA) was used to quantify the antibiotics in water. For the separation of compounds, an Agilent Zorbax Eclipse Plus C18 column (2.1 × 50 mm, 1.8 μm) was used at 30 °C. The gradient of water with 0.1% formic acid and organic phase (acetonitrile with 0.1% formic acid): 0–5.50 min 5% organic phase, 5.51–8 min 100% organic phase, 8.01–9 min 95% organic phase was applied in order to separate the compounds. The volume of the injected sample was 5 μl and the flow rate was 0.4 ml/min. An MS Agilent 6460 Triple Quad tandem mass spectrometer with an Agilent Jet Stream Electrospray Ionization interface was used in both positive and negative ion polarization using Dynamic Multiple Reaction Monitoring (dMRM) mode. Nitrogen was used as the drying gas and for collision-activated dissociation (flow rate 10 l/min). The temperature of both the drying gas and sheath gas was 350 °C. The capillary and nozzle voltage were set to 3500 V and 500 V, respectively. MassHunter software (Version 10.0, Agilent, Santa Clara, CA, USA) was used for the system control, data acquisition and data processing for UHPLC-MS. SPE recovery ranged from 9.94 to c.a. 100%^3^.

### Illumina sequencing of 16S rRNA amplicon and 16S rRNA gene sequence analysis

Water samples (1 l) were vacuum filtered through a 0.45 µm filter (Sartorius, Germany) and genomic DNA of waterborne bacteria was extracted using a Genomic Mini AX Bacteria + extraction kit (A&A Biotechnology, Poland), followed by DNA purification using Anty-Inhibitor Kit (A&A Biotechnology, Poland). The amplicon libraries of the hypervariable V3-V4 region of the 16S rRNA gene were prepared according to the 16S Metagenomic Sequencing Library Preparation Part # 15044223 Rev. B (Illumina), followed by a two-step PCR using Herculase II Fusion DNA Polymerase Nextera XT Index Kit v.2. The sample libraries were loaded on an Illumina MiSeq Platform and 2 × 300 bp reads were generated by Macrogen (South Korea).

### Bioinformatics and statistical data analyses

The 16S rRNA V3–V4 regions from the Illumina sequencing were identified by comparing the sequence reads against the Greengenes v.13 database (97% similarity, minimum score 40). The resulting sequences in the form of operational taxonomic units (OTUs) were taxonomically assigned at the phylum level or lower ranks using CLC Genomics Workbench v. 12 (Qiagen, Hilden, Germany) and Microbial Genomics Module Plugin v. 4.1. (Qiagen, Hilden, Germany).

Data obtained on the occurrence of bacterial taxa were used to calculate the relative abundance of the most common bacterial genera and phyla as well as the numbers and proportions of shared and unique bacterial genera. The calculations were conducted and graphs were constructed in R environment using the microeco package^61^.

The bacterial genera were considered as potentially containing pathogenic species if at least one species within a genus with minimum relative abundance of 0.01% was categorized as biosafety level 2 or 3 by the American Biological Safety Association^42^.

The discriminant analysis was performed in the Statistica v. 13 software (TIBCO, USA) on the OTU table in order to determine the significant differences in the abundance of bacterial groups among the study sites and sampling seasons. This was to propose the bacterial taxa that could serve as useful biomarkers of anthropogenic pressure put on the mountain river water environment.

Cluster analysis (CA) was performed in the Statistica v. 13 software (TIBCO, USA) for the study sites and sampling seasons. Euclidean distance was adopted as a measure of similarity. Ward’s agglomerative clustering was adopted as a grouping method.

Statistical differences in the number of OTUs, as the alpha-diversity measure, among samples grouped by their location along the river valley and by the season, were examined with a Kruskal–Wallis test at *p* < 0.05. Spearman’s rank correlation coefficient was calculated (*p* < 0.05) to determine the relationships between the OTU frequency and the environmental parameters.

## Supplementary Information

Supplementary Information 1.

Supplementary Information 2.

Supplementary Information 3.

Supplementary Information 4.

Supplementary Information 5.

## Data Availability

The authors declare that the data supporting the findings of this study are available within the article, in its supplementary information files as well as All high-throughput sequencing data files are available from the GenBank database (BioProject PRJNA858667).
