# Supplementary material for: Anthropogenic pollution gradient along a mountain river affects bacterial community composition and genera with potential pathogenic species
Source: Sci Rep. 2022 Oct 28;12:18140. doi: 10.1038/s41598-022-22642-x (PMC9614195; doi:10.1038/s41598-022-22642-x)
Supplement: Supplementary file 2 — Supplementary Information 2. [file 41598_2022_22642_MOESM2_ESM.docx]

Supplementary Table 1. Numbers of culturable microorganisms detected in water samples in individual dates [CFU/100 mL].

| Bacteria | *E. coli* | *E. faecalis* | coliforms | Mesophilic bacteria | Psychrophilic bacteria | *Staphylococcus* spp. | *Salmonella* | *Shigella* | Microscopic fungi |  |
| --- | --- | --- | --- | --- | --- | --- | --- | --- | --- | --- |
| unit | CFU/100 ml | | | | | | | | | |
| Sample/date | Summer | | | | | | | | | |
| GW | 0 | 0 | 0 | 0 | 5,000 | 0 | 0 | 0 | 0 |  |
| TNP | 7 | 0 | 10 | 0 | 49,000 | 300 | 0 | 0 | 0 |  |
| USTP | 15 | 3 | 25 | 10,000 | 20,000 | 200 | 0 | 0 | 100 |  |
| STP | 990,000 | 1,500 | 1,000,000 | 150,000 | 70,000 | 4,000 | 0 | 0 | 250 |  |
| DSTP1 | 10,000 | 55 | 12,000 | 15,000 | 6,000 | 500 | 0 | 0 | 0 |  |
| DSTP2 | 1,010 | 60 | 2,000 | 4,500 | 25,600 | 600 | 0 | 0 | 300 |  |
| Winter | | | | | | | | | | |
| GW | 0 | 0 | 0 | 0 | 4,000 | 100 | 0 | 0 | 0 |  |
| TNP | 0 | 3 | 0 | 0 | 40,000 | 0 | 0 | 0 | 100 |  |
| USTP | 4 | 6 | 5 | 1,200 | 20,000 | 0 | 0 | 0 | 0 |  |
| STP | 5,500 | 600 | 7,000 | 200,000 | 31,000 | 300 | 0 | 0 | 700 |  |
| DSTP1 | 4,200 | 70 | 5,500 | 10,000 | 2,500 | 1,200 | 0 | 0 | 100 |  |
| DSTP2 | 8,600 | 40 | 10,000 | 2,100 | 26,000 | 300 | 0 | 0 | 500 |  |
| Spring | | | | | | | | | | |
| GW | 0 | 0 | 0 | 0 | 1,700 | 0 | 0 | 0 | 0 |  |
| TNP | 0 | 0 | 1 | 0 | 45,000 | 1 | 0 | 0 | 0 |  |
| USTP | 0 | 0 | 2 | 3,000 | 10,000 | 0 | 0 | 0 | 0 |  |
| STP | 12 | 100 | 50 | 30,000 | 2,500 | 100 | 0 | 0 | 100 |  |
| DSTP1 | 0 | 0 | 20 | 4,000 | 1,700 | 0 | 0 | 0 | 0 |  |
| DSTP2 | 0 | 0 | 0 | 100 | 15,000 | 0 | 0 | 0 | 400 |  |

Abbreviations of the study sites are as follows: GW – groundwater; TNP – Tatra National Park; USTP – upstream of the sewage treatment plant; STP – sewage treatment plant; DSTP1 – approx.. 3 km downstream of the sewage treatment plant; DSTP1 – approx.. 7 km downstream of the sewage treatment plant.
