# Supplementary material for: Anthropogenic pollution gradient along a mountain river affects bacterial community composition and genera with potential pathogenic species
Source: Sci Rep. 2022 Oct 28;12:18140. doi: 10.1038/s41598-022-22642-x (PMC9614195; doi:10.1038/s41598-022-22642-x)
Supplement: Supplementary file 3 — Supplementary Information 3. [file 41598_2022_22642_MOESM3_ESM.docx]

**Supplementary Table 2.** Relative abundance (%) of bacterial genera with potentially pathogenic species in the water samples along the pollution gradient of the river in different sampling dates. Site abbreviations are as follows: GW – groundwater; TNP – Tatra National Park; USTP – upstream of the sewage treatment plant; STP – sewage treatment plant; DSTP1 – c.a. 3 km downstream of the STP; DSTP2 – c.a. 7 km downstream of the STP (for detailed description, please see caption of Fig. 1).

| Genus | Host | Summer | | | | | | Winter | | | | | | Spring | | | | | |
| --- | --- | --- | --- | --- | --- | --- | --- | --- | --- | --- | --- | --- | --- | --- | --- | --- | --- | --- | --- |
|  |  | GW | TNP | USTP | STP | DSTP1 | DSTP2 | GW | TNP | USTP | STP | DSTP1 | DSTP2 | GW | TNP | USTP | STP | DSTP1 | DSTP2 |
| *Enterococcus* |  | 0 | 0 | 0 | **0.03** | 0 | 0 | 0 | 0 | 0 | 0 | 0 | 0 | 0 | **0.01** | 0 | 0 | 0 | 0 |
| *Mycobacterium* |  | **6.90** | **0.01** | **0.01** | **3.75** | **0.07** | **0.05** | **1.79** | 0 | **0.05** | **0.02** | **0.03** | **0.02** | 0 | **0.01** | 0 | **1.84** | 0 | 0 |
| *Nocardia* |  | 0 | 0 | 0 | 0 | 0 | 0 | **3.32** | 0 | 0 | 0 | 0 | 0 | 0 | 0 | 0 | 0 | **0.01** | 0 |
| *Rhodococcus* |  | 0 | 0 | **0.11** | **5.73** | **0.02** | **0.08** | **1.31** | 0 | 0 | **1.36** | **0.30** | **1.61** | **0.02** | **0.01** | **0.01** | **0.20** | **0.02** | **0.02** |
| *Streptococcus* |  | 0 | 0 | 0 | **0.30** | **0.01** | **0.01** | 0 | 0 | 0 | 0 | **0.04** | **0.04** | 0 | 0 | 0 | **0.01** | 0 | 0 |
| *Clostridium* |  | 0 | 0 | 0 | **0.03** | 0 | 0 | 0 | 0 | 0 | 0 | 0 | 0 | 0 | 0 | 0 | **0.02** | **0.03** | **0.01** |
| *Burkholderia* |  | 0 | **0.05** | **0.01** | **1.49** | **0.01** | 0 | 0 | 0 | 0 | 0 | **0.03** | 0 | 0 | **0.01** | 0 | 0 | 0 | 0 |
| *Arcobacter* |  | 0 | 0 | 0 | **0.53** | **0.05** | **0.01** | 0 | 0 | **0.01** | 0 | **0.05** | **0.02** | 0 | 0 | 0 | 0 | **0.01** | 0 |
| *Escherichia* |  | 0 | **0.01** | 0 | **0.09** | **0.01** | 0 | 0 | 0 | 0 | 0 | **0.05** | **0.01** | **0.02** | **0.17** | **0.31** | 0 | **0.07** | **0.04** |
| *Stenotrophomonas* |  | 0 | **0.09** | **0.17** | **0.13** | **0.07** | **0.09** | 0 | 0 | **0.04** | **0.23** | **0.07** | **0.04** | 0 | 0 | 0 | **0.01** | **0.01** | 0 |
| *Acinetobacter* |  | 0 | **8.70** | **23.53** | **4.29** | **15.37** | **3.32** | 0 | 0 | **0.27** | **3.52** | **8.20** | **3.88** | **0.07** | **0.22** | **0.11** | **1.62** | **0.12** | **0.17** |
| Pollution gradient determinants | | | | | | | | | | | | | | | | | | | |
| No. of bacterial genera with potentially pathogenic species showing > 0.1% abundance | | 1 | 1 | 3 | 7 | 1 | 1 | 3 | 0 | 1 | 3 | 2 | 2 | 0 | 2 | 2 | 2 | 1 | 1 |
| Concentration of *E. c*oli (CFU/100 ml) [Lenart-Boroń et al. 2022] | | 0 | 7 | 15 | 990,000 | 10,000 | 1,010 | 0 | 0 | 4 | 5,500 | 4,200 | 8,600 | 0 | 0 | 0 | 12 | 0 | 0 |
| Concentration of *Staphylococcus* spp. (CFU/ml) [Lenart-Boroń et al. 2022] | | 0 | 3 | 2 | 40 | 5 | 6 | 1 | 0 | 0 | 3 | 12 | 3 | 0 | 0 | 0 | 1 | 0 | 0 |
| *E. coli*/*Staphylococcus* ratio | | - | 2.3 | 7.5 | 24,750 | 2,000 | 168.3 | 0.00 | - | 4 | 1,833.3 | 350 | 2,866.7 | - | - | - | 12 | - | - |
| Total concentration of antimicrobial agents (ng/l) | | 0.17 | 22.99 | 6.91 | 346.47 | 18.65 | 1,767.06 | 0.82 | 0.00 | 0.00 | 357.73 | 39.42 | 267.92 | 23.96 | 2.8 | 6.91 | 529.19 | 58.24 | 3.19 |

*Abundance ratio of >0.01% is indicated in bold
