# Supplementary material for: Anthropogenic pollution gradient along a mountain river affects bacterial community composition and genera with potential pathogenic species
Source: Sci Rep. 2022 Oct 28;12:18140. doi: 10.1038/s41598-022-22642-x (PMC9614195; doi:10.1038/s41598-022-22642-x)
Supplement: Supplementary file 5 — Supplementary Information 5. [file 41598_2022_22642_MOESM5_ESM.docx]

**Supplementary Legends**

Supplementary Figure 1. Changes in the bacterial community composition at the genus level along the pollution gradient. S1 – summer; S2 – winter; S3 – spring. Arrow graph shows the expected bacterial pollution gradient, darker brown shade indicates the more contaminated sites, the arrowhead indicates the course of Białka river. Site abbreviations are as follows: GW – groundwater; TNP – Tatra National Park; USTP – upstream of the sewage treatment plant; STP – sewage treatment plant; DSTP1 – c.a. 3 km downstream of the STP; DSTP2 – c.a. 7 km downstream of the STP (for detailed description, please see caption of Fig. 2).
